# Galectin-3’s Complex Interactions in Pancreatic Ductal Adenocarcinoma: From Cellular Signaling to Therapeutic Potential

**DOI:** 10.3390/biom13101500

**Published:** 2023-10-10

**Authors:** Milica Dimitrijevic Stojanovic, Bojan Stojanovic, Ivan Radosavljevic, Vojin Kovacevic, Ivan Jovanovic, Bojana S. Stojanovic, Nikola Prodanovic, Vesna Stankovic, Miodrag Jocic, Marina Jovanovic

**Affiliations:** 1 Department of Pathology, Faculty of Medical Sciences, University of Kragujevac, 34000 Kragujevac, Serbia; milicadimitrijevic@yahoo.com (M.D.S.); wesna.stankovic@gmail.com (V.S.); 2 Center for Molecular Medicine and Stem Cell Research, Faculty of Medical Sciences, University of Kragujevac, 34000 Kragujevac, Serbia; ivanjovanovic77@gmail.com; 3 Department of Surgery, Faculty of Medical Sciences, University of Kragujevac, 34000 Kragujevac, Serbia; bojan.stojanovic01@gmail.com (B.S.); ivanradoskapi@gmail.com (I.R.); nikolaprodanovickg@gmail.com (N.P.); 4 Department of Pathophysiology, Faculty of Medical Sciences, University of Kragujevac, 34000 Kragujevac, Serbia; 5 Institute for Transfusiology and Haemobiology, Military Medical Academy, 11000 Belgrade, Serbia; jocicmiodrag@gmail.com; 6 Department of Internal Medicine, Faculty of Medical Sciences, University of Kragujevac, 34000 Kragujevac, Serbia; marinna034@gmail.com

**Keywords:** Galectin-3, pancreatic ductal adenocarcinoma, prognosis, immune interaction, signaling pathways, Gal-3 inhibitors, biomarker

## Abstract

Galectin-3 (Gal-3) plays a multifaceted role in the development, progression, and prognosis of pancreatic ductal adenocarcinoma (PDAC). This review offers a comprehensive examination of its expression in PDAC, its interaction with various immune cells, signaling pathways, effects on apoptosis, and therapeutic resistance. Additionally, the prognostic significance of serum levels of Gal-3 is discussed, providing insights into its potential utilization as a biomarker. Critical analysis is also extended to the inhibitors of Gal-3 and their potential therapeutic applications in PDAC, offering new avenues for targeted treatments. The intricate nature of Gal-3’s role in PDAC reveals a complex landscape that demands a nuanced understanding for potential therapeutic interventions and monitoring.

## 1. Introduction

Pancreatic ductal adenocarcinoma (PDAC) remains one of the most lethal malignancies worldwide [1]. Its incidence has been steadily rising, and it is currently the fourth leading cause of cancer-related deaths in developed countries [2]. The 5-year survival rate remains dismally low, at around 5–10%, largely due to the fact that most cases are diagnosed at an advanced stage [3]. Several risk factors contribute to the onset of PDAC, including chronic pancreatitis, obesity, smoking, and a family history of the disease [4]. Despite extensive research, the pathogenesis of PDAC is still not fully understood, making both early diagnosis and effective treatment challenging.

The histological hallmark of PDAC is a pronounced stromal desmoplastic reaction characterized by an extensive fibrotic tissue surrounding the tumor cells [5]. This reaction involves various cellular components, including pancreatic stellate cells (PSCs), fibroblasts, immune cells, and endothelial cells, all embedded within a dense network of extracellular matrix proteins [6]. This unique stromal environment actively participates in disease progression, supporting and sustaining the malignant phenotype of the tumor [5]. It also contributes to the mechanical resistance of the tumor, leading to a reduced delivery of chemotherapeutic agents, further complicating treatment efforts [7].

PDAC is notorious for its immunosuppressive microenvironment, which plays a critical role in the tumor’s ability to evade immune surveillance [8]. This immune evasion is facilitated by a range of factors, such as the recruitment of regulatory T cells, the secretion of immunosuppressive cytokines, and the expression of inhibitory ligands [9]. Additionally, the desmoplastic stroma acts as a physical barrier, limiting the infiltration of effector T cells into the tumor site [10]. Collectively, these factors create a highly complex immune landscape in PDAC, making immunotherapeutic interventions particularly challenging [11].

Galectins are a multifaceted family of proteins recognized for their ability to bind β-galactosides through carbohydrate-recognition domains (CRDs) [12]. Structurally, they are divided into three distinct subtypes: prototype galectins, tandem repeat galectins, and the unique chimeric galectin [13]. Prototype galectins, such as galectin-1, -2, -5, -7, -10, -11, -13, -14, and -15, consist of a single CRD capable of forming homodimers, while tandem repeat galectins, including galectin-4, -6, -8, -9, and -12, are composed of two CRDs connected by a linker region [13]. These proteins are involved in critical cellular processes like mitosis, apoptosis, cell cycle progression, immune system regulation, cellular proliferation, migration, adhesion, and carcinogenesis [14,15]. Particularly noteworthy among the galectins is Galectin-3 (Gal-3), a chimeric type characterized by a CRD fused with proline- and glycine-rich stretches [16]. Its distinct structure and binding specificity to glycoconjugates containing β-galactose and N-acetyllactosamine (LacNAc) lead to a broad range of applications and investigations, highlighting crucial functions in biological activities such as immune response, inflammation, cancer development, apoptosis, cell migration, and adhesion [16,17,18].

In this review, we will explore the complex roles of Gal-3 within the challenging context of pancreatic ductal adenocarcinoma. We will delve into Gal-3’s unique structure and binding specificities, examining its multifaceted involvement in critical biological processes such as immune response modulation, inflammation, and cancer development. Special attention will be given to the interactions of Gal-3 with PDAC’s characteristic stromal desmoplastic reactions and immunosuppressive environment. We aim to elucidate how targeting Gal-3 could lead to novel therapeutic strategies to tackle the longstanding challenges in PDAC treatment. Through a comprehensive analysis of current research, this review seeks to highlight the potential of Gal-3 as both a valuable scientific investigation target and a promising avenue for therapeutic innovation in one of the most formidable cancers.

## 2. Galectin-3: Structure and Function in Cellular Biology

Galectin-3 is a notable β-galactoside-binding protein of approximately 31 kDa, uniquely structured with three domains [19]. These domains include a distinctive N-terminal domain responsible for translocation and secretion, a carbohydrate recognition domain specific to the C-end for N-acetyl galactosamine binding, and a repeat region abundant in proline, glycine, and tyrosine [19]. The Gal-3 structure also comprises an NH2-terminal domain of 12 amino acids, an alpha-collagen-like sequence of 110 amino acids, and a COOH-terminal CRD with 130 amino acid residues [20]. Within the C-terminal domain, the Asparagine–Tryptophan–Glycine–Arginine (NWGR) anti-death motif, highly conserved among B-cell lymphoma 2 (Bcl-2) family proteins, functions as an anti-apoptotic molecule [21].

Gal-3 demonstrates multifaceted biological roles and is essential for various cellular functions. It is involved in cell–cell adhesion, cell–matrix interaction, macrophage activation, angiogenesis, metastasis, apoptosis, cell growth, differentiation, migration, and cancer drug resistance [16,18]. In the cytoplasm, Gal-3 functions as an apoptosis inhibitor and regulates cytoplasm–nucleus trafficking, while in the nucleus, it acts as an mRNA-splicing promoter [22,23].

The NWGR motif in Gal-3 plays a crucial anti-apoptotic role [24]. This motif effectively shields cancer cells from the apoptosis-inducing effects of agents like cisplatin, etoposide, Tumor Necrosis Factor-alpha (TNF-alpha), and nitric oxide [25,26,27]. Furthermore, in mice lacking Gal-3, peritoneal macrophages exhibited an enhanced susceptibility to apoptosis [28]. Gal-3 also inhibits apoptosis in T-cells and epithelial cells, contributing to resistance against anticancer drug-induced apoptosis, especially in pancreatic cancer cells [29,30,31].

Gal-3 on tumor cell surfaces acts as an adhesion molecule, promoting cell-to-cell and cell-to-matrix connections [32]. It binds primarily to extracellular glycoproteins through terminal galactose residues, though it has a higher affinity for certain galactose structures [30]. This extracellular role is believed to support immune cell movement and metastasis [30,33].

Galactin-3 is a versatile protein with a wide array of functions that vary depending on its cellular location. From aiding in cell-to-cell interactions on the cell membrane to intricate roles within the cytoplasm and nucleus, its multifaceted roles are pivotal in cellular processes. Table 1 provides a summarized overview of the biological functions of Gal-3 in the cell membrane, cytoplasm, and nucleus.

### Galectin-3 in Various Carcinomas: Expression, Metastasis, and Prognostic Implications

Galectin-3 (Gal-3) serves as a pivotal molecule in the intricate landscape of oncology, with expression patterns spanning a multitude of malignancies, including gastrointestinal, cardiovascular, urinary, respiratory, breast, melanoma, and thyroid systems [34,35,36,37,38,39,40,41,42]. Its role in cancer biology is not merely a byproduct of its widespread distribution but rather an indication of its intimate involvement in critical cellular processes.

In the realm of metastasis, Gal-3 exerts significant influence [30]. Specifically, in breast and prostate neoplasms, it is implicated in modulating cell detachment, fine-tuning cell-to-cell and cell-ECM interactions, thereby enhancing cellular motility and invasion capacities [24]. For instance, metastatic breast cancer cells displaying elevated Gal-3 expression manifest augmented adherence capacities to endothelial cells [43]. Conversely, a downregulation of Gal-3 correlates with diminished cell motility observed in specific malignancies like colon cancer and glioblastoma, underscoring its regulatory role in metastatic pathways [44,45].

Regarding prognostic implications, the expression dynamics of Gal-3 across carcinomas present a complex picture. While overexpression in certain malignancies like hepatocellular carcinoma signals a grim prognosis, its diminished presence in others, such as ovarian and breast cancer, indicates potential adversities [46,47,48]. Colorectal cancer offers a particularly perplexing paradigm, with contrasting studies presenting both a high and low expression of Gal-3 as potential harbingers of poor prognosis [44].

Lastly, the functional implications of Gal-3 are expansive, as in vitro analyses unveil its capability to modulate diverse cellular outcomes. From impeding cell migration in prostate malignancies to potentiating apoptosis in gastric neoplasms, the spectrum of its influence is vast [24,49].

## 3. Galectin-3 Expression in Pancreatic Ductal Adenocarcinoma: A Comprehensive Overview

In the complex landscape of pancreatic cellular physiology, Galectin-3 (Gal-3) holds a pivotal position, and its differential expression provides critical insights into its multifaceted roles. In physiological pancreatic tissue, Gal-3 predominantly localizes to the ductal cells [50]. Notably, a substantial subset of these ductal cells showcases pronounced cytosolic or combined nuclear staining, underscoring the integral presence of this protein. Meanwhile, acinar cells present a more restrained picture, with only sporadic instances of nuclear Gal-3 expression, suggesting a more subdued functional role [50].

Transitioning from physiological to pathological settings, the expression of Gal-3 amplifies markedly. Pathologies such as chronic pancreatitis and pancreatic cancer demonstrate a near-universal and augmented expression of this molecule, with over 95% of cells expressing it [50]. Contrastingly, its absence in specific neoplasms, notably PNEN and GIST, offers a diagnostic differential, elucidating its potential significance in discerning these malignancies [51]. The expression of Gal-3 in normal pancreatic tissue and various pathological states is summarized in Table 2.

A deeper exploration of pancreatic carcinoma tissues reveals a predominant cytoplasmic overexpression of Gal-3, with nuclear staining being notably subdued [50]. This upregulation, corroborated both at the mRNA and protein strata through rigorous methodologies like Northern and Western blotting indicates a profound pathophysiological role [52]. The pronounced overexpression in the majority of pancreatic cancer cell lines further emphasizes its significance in neoplastic transformation [52,53].

The intracellular dynamics of Gal-3 in pancreatic tumor cells are characterized by a phenomenon termed nucleocytoplasmic shuttling [54]. Its nuclear variant is implicated in processes encompassing gene transcription and pre-mRNA splicing. Simultaneously, the cytoplasmic iteration plays an instrumental role in averting apoptosis and endorsing processes such as cell proliferation, differentiation, and survival. This is orchestrated through pivotal interactions with molecular entities like Kirsten rat sarcoma viral oncogene homolog (K-ras) and protein kinase B (Akt), underscoring the intertwined and expansive molecular networks in which Gal-3 is embroiled [54].

Remarkably, Gal-3 manifests prominently in early-stage primary tumors, particularly those without lymph node involvement, emphasizing its prospective role in oncogenic processes [53]. Yet, as the carcinoma advances, exhibiting metastatic proclivities, Gal-3 expression in primary tumors appears to recede. This intricate modulation, juxtaposed with its heightened expression in metastatic sites such as lymphatic nodes and liver tissues, underscores Gal-3’s pivotal role, spanning from oncogenesis to facilitating metastatic evolution. Such patterns suggest that Gal-3 may not only drive tumor initiation but could also support metastatic expansion, potentially aiding malignant cells in their dissemination and endurance [53].

### Galectin-3 in Pancreatic Ductal Adenocarcinoma: Diagnostic and Prognostic Implications

In the arena of pancreatic cancer biomarkers, Galectin-3 (Gal-3) stands out as a molecule of considerable significance. Predominantly expressed in pancreatic cancer tissues, its presence in peripheral blood offers a unique window for disease diagnosis and prognosis [55]. Elevated serum Gal-3 levels, particularly in metastatic disease, delineate a clear demarcation from benign pancreatic conditions [55].

For pancreatobiliary neoplasms, Gal-3 boasts a sensitivity of 88.9% and specificity of 60.0% at a threshold of 6.2 ng/mL [56]. Its combined assessment with markers such as Carcinoembryonic Antigen (CEA) and Carbohydrate Antigen 19-9 (CA19-9) augments its diagnostic potency. Notably, a rise in Gal-3 levels correlates with poorer outcomes, establishing its prognostic merit in biliary and pancreatic malignancies [56].

Post-operative evaluations underscore Gal-3’s clinical utility. A marked reduction in Gal-3 levels after radical excision signals a favorable trajectory, whereas a surge or static level predicts carcinoma recurrence [55]. Nevertheless, while Gal-3 emerges as a promising biomarker, its association with macrophages, mast cells, and eosinophils coupled with its involvement in inflammatory processes necessitate meticulous interpretation in clinical contexts [56,57]. In this light, emerging data highlighting the potential of galectin-binding glycoproteins to enhance Gal-3’s diagnostic accuracy further underscore the complexity and promise of its application [57].

In summation, while Galectin-3 heralds a promising horizon in pancreatic cancer diagnostics and prognostics, rigorous studies are imperative to delineate its definitive clinical utility.

## 4. Galectin-3 in Pancreatic Carcinoma: Apoptosis Resistance and Drug Sensitivity

Pancreatic adenocarcinoma demonstrates a pronounced resistance to many anticancer drugs, often attributed to its enhanced resistance to apoptosis [58]. One protein, Gal-3, has been increasingly recognized as a key factor in this resistance mechanism in PDAC [59].

Evidence from in vitro studies underscores the role of Gal-3 in apoptosis resistance. When Gal-3 is silenced using siRNA, cells show a higher rate of apoptosis in response to chemotherapy drugs like gemcitabine (GEM) and Cisplatin [59]. Interestingly, the mechanism appears to involve the mitochondrial pathway. After Gal-3 silencing, an increase in mitochondrial depolarization is observed following GEM treatment, leading to the activation of caspase-9, a central player in mitochondrial apoptosis. Notably, this effect does not extend to the extrinsic apoptosis pathway, as evidenced by the lack of change in caspase-8 activity [59].

Support from in vivo studies strengthens these findings. In mouse models, tumors treated with Gal-3 siRNA showed increased sensitivity to GEM, resulting in slowed tumor growth [59]. Delving into the underlying mechanisms, it is suggested that the role of Gal-3 in apoptosis may relate to its connection with the NWGR anti-death motif in the Bcl-2 protein family. Furthermore, Gal-3 can influence the Mitogen-Activated Protein Kinase (MAPK) pathways, including the Extracellular Signal-Regulated Kinase (ERK) and the c-Jun N-terminal Kinase (JNK). Both pathways have previously been shown to play roles in apoptosis resistance across various cancers when exposed to chemotherapy agents [60].

## 5. Galectin-3 in Intracellular Signaling Pathways: Interactions and Implications in Pancreatic Cancer

Gal-3, a crucial regulator in cellular processes, modulates various signaling pathways, one of which is the Epidermal Growth Factor Receptor (EGFR)/AKT/ Forkhead Box O3 (FOXO3) pathway [61]. It has been elucidated that the interaction between Gal-3 and this pathway, along with others such as EGFR/ERK/ Runt-related Transcription Factor 1 (Runx1) and Bone Morphogenetic Protein (BMP)/smad/ Inhibitor of DNA Binding 3 (Id-3), plays pivotal roles in PDAC progression [61,62,63]. However, further studies are needed to consolidate these findings with other research, and a deeper understanding of the nuances involved is essential.

Intracellularly, Gal-3 is dynamic and translocates between the nucleus and cytoplasm, mediating intricate associations with an array of intracellular ligands. Among these are BCL-2, β-catenin, Kirsten Rat Sarcoma Viral Oncogene Homolog (K-RAS) GTP, Thyroid Transcription Factor 1 (TTF1), Mucin 1 (MUC1)/EGFR, and Activator Protein 1 (AP-1) [64]. Interestingly, its association with components of the AP-1 transcription factor complex, such as c-Jun and Fra-1, has implications in colon cancer. Furthermore, in certain cancers, Gal-3 enhances wnt/β-catenin signaling and activates Ras, Ras-Like Protein A (RalA) signaling, underscoring its comprehensive role [64].

One prominent relationship is between Gal-3 and oncogenic K-Ras in PDAC. There is a significant elevation of Gal-3 in both human and mouse PDAC cells, which has been implicated in K-Ras activation [65]. It is noteworthy to mention that K-Ras mutations are potent drivers of PDAC [66]. The molecular interactions of Gal-3 with K-RAS GTP, particularly its role in relocating the protein to the plasma membrane and its significance in nanoclusters, suggest its role in PDAC cell behaviors like proliferation and invasion. Moreover, this interaction influences Ras activity in a complex manner, potentially offering therapeutic points of intervention [65].

Another pathway influenced by Gal-3 is the Wingless/Int-1 (Wnt) signaling pathway, especially concerning the invasion and migration of pancreatic cancer cells [67]. The molecular intricacies reveal that Gal-3 silencing impacts β-catenin and other key molecules, ultimately influencing cell invasion. Moreover, Gal-3 modulates the expression of significant molecules connected to invasion, solidifying its pivotal role [67].

The interplay between Gal-3 and Mucin 1 (MUC1) in the context of PDAC is critically significant. Aberrant interactions of MUC1 with certain proteins, notably EGFR, culminate in oncogenic signaling [68]. The intricate involvement of Gal-3 in this realm underscores its profound influence on the EGFR-MUC1-Ras-Raf-ERK signaling axis. Specifically, Gal-3’s modulation of this pathway contributes to PDAC progression [68]. Beyond its direct involvement in this signaling cascade, Gal-3 demonstrates a capacity to stabilize MUC4 mRNA, hinting at its broader regulatory role in the cellular landscape [69].

In essence, the multi-dimensional influence of Gal-3 on various signaling pathways and cellular processes emphasizes its significance in PDAC. The extensive interactions and underlying mechanisms highlight the need for a more in-depth exploration and offer potential avenues for targeted therapeutic interventions in pancreatic cancer.

## 6. Galectin-3’s Interaction with the Immune Response in Pancreatic Cancer: A Dual Role

Galectin-3 is a unique beta-galactoside-binding protein that plays a significant role in the development of acute pancreatitis [70]. Its complex interaction with the immune system in PDAC presents a dual role in immune regulation, playing crucial roles in both immune evasion and the maintenance of immune stability [71].

In the PDAC tumor microenvironment, Gal-3 plays a pivotal role in fostering an immunosuppressive milieu [72,73]. It engages with immune cells, influencing their function, and is instrumental in dampening the anti-tumor response of certain T cells [73]. Furthermore, Gal-3 has been identified as a factor facilitating tumor immune evasion, impacting various immune cell types and pathways, including interactions with recognized immune checkpoints and promoting T-cell apoptosis [29,71].

**Figure 1:**
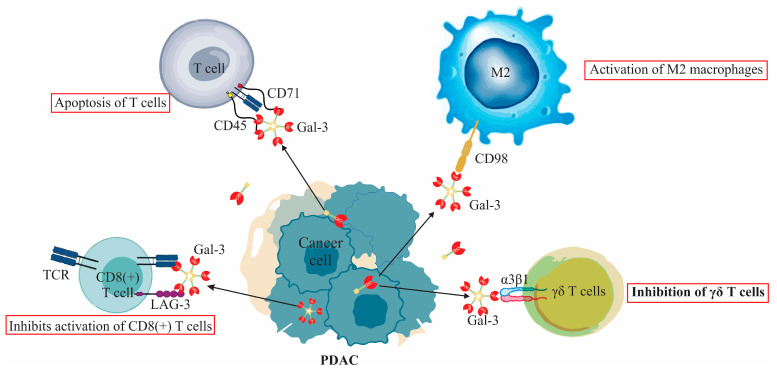
Extracellular functions of Galectin-3 (Gal-3) in PDAC immune modulation: The figure delineates Gal-3’s suppressive interaction with γδ T cells, emphasizing its binding to α3β1 integrin. It further illustrates Gal-3’s role in tumor immune evasion, showcasing its dual interactions as an immune checkpoint with LAG-3 and its induction of T-cell apoptosis via CD45 and CD71. Additionally, the figure highlights the pivotal role of Gal-3 in M2 macrophage activation, emphasizing its interaction with CD98 leading to PI3K activation. A distinct segment showcases Gal-3 constraining TCR mobility on anergic T lymphocytes, which translates to the inhibition of CD8(+) T cell activation.

Interestingly, Gal-3’s impact on immune regulation is not solely suppressive. The protein displays a contrasting intracellular function where it promotes T-cell proliferation and inhibits apoptosis, counteracting its extracellular immunosuppressive activities (Figure 1). This dual role underscores the complexity of Gal-3’s interaction with the immune response in pancreatic cancer [71].

### 6.1. Galectin-3’s Interaction with T Lymphocytes and Its Role in Pancreatic Ductal Adenocarcinoma

Galectin-3 plays a multifaceted role in the pathogenesis and progression of pancreatic ductal adenocarcinoma, and its interaction with T lymphocytes represents a vital aspect of this complexity [73]. T lymphocytes, as part of the adaptive immune system, also play significant roles in other pancreatic pathologies, primarily in acute pancreatitis [74].

In the tumor environment, PDAC cells notably express and release Gal-3, leading to various immunomodulatory effects [73]. Notably, when Gal-3 is present in an extracellular manner, it hampers the mobility of the T-cell receptor (TCR), thus leading to the emergence of anergic T lymphocytes [29]. This alteration plays a key role in diminishing T cell-mediated immune activities, paving the way for the expansion of the tumor [29]. A prominent manifestation of this inhibitory effect is seen in the suppression of the activation of CD8(+) T cells. This phenomenon provides an explanation for the characteristic immunosuppressive environment observed in PDAC [64].

The release of Gal-3 is significantly enhanced when PDAC cells are co-cultured with γδ T cells [73]. This interaction is noteworthy for its resultant impediment on the proliferation of γδ T cells, with the Vδ2 subtype being particularly affected [73]. An intriguing facet of this observation is that the binding of Gal-3 to the glycosylated surface receptor α3β1 integrin of γδ T cells is instrumental in this inhibitory effect. However, it is of importance to note that this interaction does not compromise the cytotoxic potential of the T cells [73].

Furthermore, Gal-3’s binding to the β3β1 integrin on T cells hinders their proliferation, which contributes to the immunosuppressive environment seen in PDAC [71]. When outside the cell, Gal-3 can induce T cell apoptosis and weaken the immune response by binding with specific receptors, including CD71 and CD45 [71]. It is also observed that higher Gal-3 levels in pancreatic tumors are linked with decreased activity of CD8(+) T lymphocytes, mainly because of reduced IFN-gamma secretion. Conversely, when Gal-3 is absent on CD4(+) T cell surfaces, there’s an increase in IFN-gamma production by these lymphocytes [71].

Contrastingly, inside the cell, Gal-3 has different roles in the immune system. It works against the apoptosis process, helps more T cells to grow, and makes balance in autoimmunity by reducing too much inflammation [29]. It is also worth mentioning that this protein, when working with Bcl-2, prevents T cell from undergoing apoptosis [29].

### 6.2. Galectin-3’s Interaction with Macrophages and Its Role in Pancreatic Ductal Adenocarcinoma

Galectin-3 is prominently involved in macrophage differentiation and activation within the context of pancreatic ductal adenocarcinoma [71]. Upon engagement with the glycoprotein receptor CD98, Gal-3 instigates PI3K activation. Such interaction not only bolsters the proliferation of M2 macrophages but also encourages the phenotypic shift from M1 to the immunosuppressive M2 subtype [75]. This pronounced activation of M2 macrophages, driven by Gal-3, augments immune evasion capabilities of PDAC cells, and the concomitant systemic immunosuppression contributes to the advancement of PDAC [71].

## 7. Galectin-3 in the Tumor Microenvironment of Pancreatic Ductal Adenocarcinoma

The role of Gal-3 within the tumor microenvironment (TME) of pancreatic ductal adenocarcinoma is multifaceted and not yet fully elucidated. It serves as a key mediator in cellular signaling by engaging with glycoconjugates of receptors on the cell surface, contributing to various mechanisms that underlie tumor progression [64].

Gal-3 has been found to support tumor cell adaptation and survival in the stressful conditions of the TME by activating several cellular signaling pathways, such as RAS/MAPK, Nuclear Factor-kappa B (NF-kB), and Focal Adhesion Kinase (FAK)/Vascular Endothelial Growth Factor (VEGF) [76]. These pathways are implicated in various aspects of tumor biology, including growth, survival, inflammation, and angiogenesis [64]. Circulating Gal-3 has been associated with the induction of metastasis-promoting cytokines from vascular endothelium, indicating a potential role in the spread of cancer cells to distant sites [55].

In conclusion, Gal-3’s interactions and signaling within the TME of PDAC are complex and multifunctional, impacting various facets of tumor growth, survival, and metastasis. Understanding these intricate mechanisms offers the potential for developing targeted interventions aimed at modifying the tumor environment, thus opening new avenues for therapeutic strategies in the management of PDAC.

### 7.1. Galectin-3 Interaction with Pancreatic Stellate Cells (PSCs) and Cancer-Associated Fibroblasts (CAFs) in PDAC

In pancreatic ductal adenocarcinoma, Gal-3 has a profound influence on pancreatic stellate cells, mediating critical tumor–stroma interactions [64]. Secreted Gal-3, including its recombinant form (rGal-3), has been demonstrated to stimulate PSC proliferation, as evidenced by the studies involving conditioned media derived from cells with enhanced Gal-3 expression [64]. This effect is not merely restricted to promoting PSC proliferation; it also magnifies their migratory and invasive attributes [64]. Additionally, Gal-3 is associated with the upregulation of cellular markers such as alpha-smooth muscle actin (α-SMA) and key components of the extracellular matrix (ECM), signifying its contributory role in PSC activation. Concomitantly, Gal-3 modifies the inflammatory cascade, as corroborated by the escalated secretion of pro-inflammatory cytokines from Gal-3-treated PSCs, thus potentiating the advancement of PDAC [64].

Transitioning from its influence on PSCs, Gal-3 is also discerned to substantially modulate cancer-associated fibroblasts (CAFs), salient components of the PDAC stromal framework [64]. This protein plays a crucial role in mediating interactions between CAFs and neoplastic cells, leading to enhanced CAF proliferation, invasion, and upregulated expression of α-SMA and ECM proteins [64].

In summary, GAL-3’s intricate interactions with PSCs and CAFs are fundamental to the pathogenesis and progression of PDAC, highlighting its multifaceted role within the complex tumor microenvironment. Detailed interactions between GAL-3 and both PSCs and CAFs are presented in Table 3.

### 7.2. Galectin-3 Interaction with Ischemia and Nutrient Deprivation in PDAC

In pancreatic ductal adenocarcinoma, Gal-3 plays a significant role in responding to ischemia and nutrient deprivation, conditions commonly found in the tumor microenvironment [61]. The expression of Gal-3 is modulated by these conditions, as evidenced by increased Gal-3 mRNA and protein levels in pancreatic cancer cell lines, such as PANC-1 and Mia PaCa-2, when exposed to hypoxic and nutrient-deprived environments [61].

This increase in Gal-3 expression is intricately tied to the metabolic reprogramming of tumor cells, often termed the “Warburg effect” [77]. Here, tumor cells shift their metabolic processes to favor glucose metabolism through glycolysis over oxidative phosphorylation [77]. Gal-3 potentially regulates this shift, resulting in alterations in key molecules like glucose transporter1 (GLUT-1), AMP-activated protein kinase (AMPK), and mammalian target of rapamycin (mTOR). By enhancing aerobic glycolysis and enzyme activities, Gal-3 ensures the necessary energy levels for cell survival in a deprived environment [61].

In addition to its role in metabolic adaptation, the increased secretion of Gal-3 in nutrient-deprived conditions is linked to several other functions that help maintain pancreatic cancer cell viability and homeostasis. These include angiogenesis enhancement, immune modulation, and the uptake of extracellular content. Through the regulation of vascular availability and nutrient competition with immune cells, secreted Gal-3 contributes to the survival of PDAC cells in hostile microenvironments [61].

The hypoxic and nutritionally deprived conditions in PDAC further stimulate Gal-3 expression, which reduces lymphocyte infiltration, thereby facilitating tumor cell development. The inhibition of Gal-3 has been found to have a substantial anti-tumor effect, leading to apoptosis and decreased angiogenesis and invasion [71]. These findings suggest that hypoxia and nutritional deprivation influence Gal-3 expression in PDAC as part of an adaptive response to ensure tumor cell survival.

## 8. Galectin-3 Binding Protein in Pancreatic Ductal Adenocarcinoma: Function, Regulation, and Therapeutic Potential

Galectin-3 binding protein (Gal-3BP) is a significant player in pancreatic ductal adenocarcinoma progression, involved in various cellular pathways that contribute to tumorigenesis [78]. Understanding its role in PDAC sheds light on potential therapeutic interventions and helps to elucidate the complexity of tumor development [78].

A key aspect of Gal-3BP’s role in PDAC involves enhancing galectin-3-mediated epidermal growth factor receptor (EGFR) signaling. This activity stimulates the increase in cellular Myelocytomatosis oncogene (cMyc), a cell proliferation regulator, and facilitates the epithelial-mesenchymal transition (EMT), a vital process in cancer metastasis. Research demonstrates the necessity of Gal-3BP for PDAC cell growth both in vitro and in vivo [78].

Manipulating the expression of Gal-3BP yields insights into its functional roles. Stable knockdown of Gal-3BP results in a significant reduction in the growth and tumorigenic potential of PDAC cells, with a corresponding decrease in adhesion and migration capabilities [78]. EMT markers like Zinc Finger E-box-Binding Homeobox 1 (Zeb1), Claudin-1, and Snail are also downregulated. In contrast, overexpression of Gal-3BP leads to the upregulation of EMT markers N-cadherin, Snail, and Zeb1, particularly in primary PDAC cells that naturally exhibit low levels of Gal-3BP [78].

The therapeutic potential of targeting Gal-3BP has been explored, with administration of Gal-3BP antibodies inhibiting EGFR-Myc signaling and metastasis of PDAC cells in patient-derived preclinical models. This evidence positions Gal-3BP as a viable target for therapeutic intervention [78].

Further insights into Gal-3BP’s role come from its overexpression in PDAC tumor tissue and the abnormal N-glycosylation levels of circulating Gal-3BP in PDAC patients [79]. This elevation in N-glycosylated Gal-3BP peptides is typically seen in localized stage II disease and may correlate with the cancer neoplasm size. The abnormal change in N-glycosylation levels at an early stage of PDAC progression may provide significant insights into complex glycosylation events associated with cancer metabolic reprogramming, glycan biosynthesis, and immune response, all driving PDAC tumorigenesis [79]. In Table 4, the main aspects of Gal-3BP’s function, regulation, and therapeutic potential in PDAC are summarized.

## 9. Galectin-3 in PDAC: Therapeutic Implications and Future Endeavors

Gal-3’s intricate role in the pathogenesis of PDAC has ushered in a new era of therapeutic contemplations. By modulating the activities of Gal-3, one stands at the precipice of altering fundamental oncogenic processes, encompassing neoplastic proliferation, metastasis, angiogenesis, and the intricate dance of immune evasion [33].

Delving deeper into the domain of polysaccharide inhibitors, one cannot overlook the significance of HH1-1, a distinguished homogeneous polysaccharide. Its affinity for Galectin-3 impedes the subsequent interactions with EGFR, culminating in an obstruction of the Galectin-3/EGFR/AKT/FOXO3 signaling cascade [62]. Such molecular interferences herald a cascade of cellular consequences: a halt in cell proliferation, initiation of the apoptotic pathways, and a reduction in the faculties of cellular migration, invasion, and angiogenesis. Furthermore, through subtle modulations in the FOXO3 transcriptional landscape, HH1-1 orchestrates a diminution in both Galectin-3 and EGFR expression, earmarking it for further therapeutic exploration [62].

Equally of note is the Modified Citrus Pectin (MCP), another molecular entity challenging Galectin-3’s hegemony. Preliminary investigations in preclinical models advocate for its efficacy in mitigating tumor vigor, aligning its therapeutic potential with other contenders in the field [64].

In the domain of polysaccharides, RN1, derived from the Panax notoginseng flower, emerges as a notable molecule with potential therapeutic implications for PDAC [63]. This compound demonstrates a marked ability to downregulate Gal-3 expression, consequently attenuating PDAC cell proliferation. It exerts its effect primarily by disrupting the interactions of Gal-3 with its associated receptors, thus inhibiting key oncogenic pathways, namely EGFR/ERK/Runx1, BMP/smad/Id-3, and integrin/FAK/JNK [63]. Coupled with its benign toxicity profile, RN1 holds promise for more extensive research in the context of PDAC therapeutics [63].

Within the evolving landscape of therapeutic advancements, there emerges a sophisticated approach focusing on precision-targeted strategies, capitalizing on the specificity of antibodies or the intricate mechanisms of RNA interference against Gal-3 [63]. Current scientific pursuits are gravitating towards the judicious identification of molecular epitopes and the optimization of their delivery modalities. In tandem with these approaches, there is a burgeoning interest in combination therapies. Augmenting the therapeutic salience could well be realized by juxtaposing Gal-3 inhibitors with an array of other modalities such as immune checkpoint inhibitors, chemotherapeutic agents, or targeted therapies. The pursuit of such synergies necessitates a profound understanding of their interplay and the crafting of methodical clinical exploration blueprints [63].

Moreover, the domain of therapeutic vaccines offers new avenues for exploration [80]. Influenced by models like GCS-100, the direction of investigation might pivot towards the development of vaccines adept at stimulating the immune response against cells presenting Gal-3 [80]. The amalgamation of these therapeutic vaccines with agents such as GCS-100 may potentially amplify the efficacy of tumor-infiltrating lymphocytes, ultimately leading to augmented cytotoxic effects [81]. Additionally, Table 5 provides a comprehensive synthesis of these therapeutic strategies, aiding in a clear and organized understanding of the research landscape surrounding Gal-3 targeting in PDAC.

## 10. Future Directions in Researching Gal-3 in PDAC

The intricate relationship between Gal-3 and the pathogenesis of pancreatic ductal adenocarcinoma has gained significant attention in recent years, opening the door to a myriad of research opportunities. While we have made substantial strides in understanding its mechanistic role in PDAC, several promising avenues remain to be explored.

Firstly, an in-depth investigation into the molecular pathways influenced by Gal-3 in PDAC can pave the way for more targeted therapeutic strategies. This involves not only understanding its role in tumor cell growth and metastasis but also its interaction with the tumor microenvironment, immune modulation, and other relevant signaling cascades.

Additionally, while initial studies have highlighted the potential of targeting Gal-3 directly, a more holistic approach could consider the broader network of interacting proteins and molecules. By understanding the wider interactome of Gal-3 in PDAC, there lies potential for the identification of novel drug targets or combinatory therapeutic approaches. Such studies can benefit from advanced molecular techniques, including proteomics and high-throughput screening methods.

Furthermore, the translational implications of Gal-3 research should not be overlooked. As more preclinical findings emerge, a structured transition into clinical trials will be imperative. Establishing robust animal models that mimic human PDAC can serve as crucial platforms for assessing the safety and efficacy of Gal-3-targeted interventions. Parallel to this, patient-derived xenografts and organoid models may provide valuable insights into patient-specific responses, facilitating personalized therapeutic strategies.

Lastly, with the increasing recognition of the tumor microenvironment’s role in PDAC, elucidating the spatial and temporal dynamics of Gal-3 expression within this milieu might yield invaluable insights. This can be complemented by advanced imaging techniques and computational modeling to predict and monitor tumor progression and response to therapy.

In summary, the future of Gal-3 research in PDAC appears promising, with numerous paths to explore. Collaborative interdisciplinary efforts will undoubtedly play a pivotal role in advancing our understanding and ultimately translating findings into clinical benefit.

## 11. Conclusions

In conclusion, Galectin-3 (Gal-3) stands out as a pivotal and multifaceted molecule in the intricate tapestry of pancreatic ductal adenocarcinoma (PDAC) pathogenesis and progression. Its diverse roles span from orchestrating cellular interactions within the tumor microenvironment, mediating oncogenic signaling cascades, to modulating immune responses. The dual nature of Gal-3’s influence, fostering both immunosuppressive conditions while maintaining immune stability, speaks to the protein’s profound complexity. Elevated serum levels of Gal-3 signify its potential as a diagnostic and prognostic tool, differentiating malignant from benign pancreatic conditions. Moreover, its function in apoptosis resistance and involvement in various intracellular signaling pathways further spotlight its promise as a therapeutic target. Understanding the manifold interactions and dynamics of Gal-3 in PDAC is paramount. As we confront the formidable challenges presented by PDAC, our continued investigation into the multifarious aspects of Gal-3 is crucial. Only with a deeper comprehension can we harness its potential, paving the way for innovative therapeutic avenues and enhancing patient outcomes in the face of this aggressive malignancy.

## Figures and Tables

**Table 1:** Biological roles of Galectin-3.

Location/Function	Activity or Role
Cell–cell Adhesion	Involved in various cellular functions
Cytoplasm	Functions as an apoptosis inhibitor, regulates cytoplasm–nucleus trafficking
Nucleus	Acts as an mRNA-splicing promoter
Surface of Tumor Cells	Functions as an adhesion molecule, facilitates cell interactions
Extracellular Function	Aids immune cell migration and metastasis

**Table 2:** Galectin-3 expression in different pancreatic conditions.

Condition	Gal-3 Expression	Notes
Normal Pancreatic Tissue	Localized to ductal cells	Weaker expression in acinar cells
Chronic Pancreatitis	Over 95% expression	More uniform
Pancreatic Cancer	Over 95% expression	Mainly cytoplasmic, with weak nuclear staining
Pancreatic Neuroendocrine Tumors	Undetectable	Differentiation aspect in diagnosis
Gastrointestinal Stromal Tumors	Undetectable	Differentiation aspect in diagnosis

**Table 3:** Detailed interactions of Galectin-3 with pancreatic stellate cells and cancer-associated fibroblasts in PDAC.

Category	Interaction with	Mechanisms and Actions	Impact on PDAC
Pancreatic Stellate Cells (PSCs)	- Proliferation	- Secreted Galectin-3, including recombinant form, stimulates growth	Encourages tumor-stroma interactions; promotes PDAC progression
	- Migration and Invasion	- Galectin-3 overexpression encourages migration	Increases potential for metastasis and invasion of surrounding tissues
	- Activation	- Enhances alpha-smooth muscle actin (α-SMA) and extracellular matrix (ECM) proteins	Facilitates PSC activation; contributes to fibrotic environment
	- Inflammatory Response	- Influences secretion of pro-inflammatory cytokines	Modulates inflammation, contributing to a favorable tumor microenvironment
Cancer-Associated Fibroblasts (CAFs)	- Interaction with Tumor Cells	- Mediates essential connections between CAFs and tumor cells	Enhances connectivity between cellular elements within the tumor environment
	- CAF Proliferation and Invasion	- Encourages CAF growth and invasion through interaction with α-SMA and ECM proteins	Strengthens the supportive stromal network aiding in tumor growth and development
	- α-SMA and ECM Expression	- Induces the expression of α-SMA and ECM proteins within CAFs	Reinforces the structural integrity of the tumor, facilitating progression and survival

**Table 4:** The main aspects of Gal-3BP’s function, regulation, and therapeutic potential in PDAC.

Aspect	Description
Role in PDAC	Involved in tumorigenesis, enhances Gal-3-mediated EGFR signaling, and stimulates the EMT process.
Effect on cMyc and EMT	Stimulates the increase in cMyc; facilitates EMT, affecting cancer metastasis.
Influence on Cell Growth	Essential for PDAC cell growth both in vitro and in vivo.
Manipulation of Expression	- Knockdown: reduction in growth, adhesion, and migration; downregulates EMT markers.
	- Overexpression: upregulates EMT markers like N-cadherin, Snail, and Zeb1.
Interaction with EGFR	Noteworthy interaction with EGFR; upregulation of cMyc via EGFR activation.
Therapeutic Potential	Targeting with Gal-3BP antibodies inhibits EGFR-Myc signaling and metastasis in preclinical models.
Expression in Tumor Tissue	Overexpression in PDAC tumor tissue; abnormal N-glycosylation levels in circulating Gal-3BP.
N-Glycosylation in Stage II	Elevation correlates with cancer neoplasm size; insights into glycan biosynthesis and immune response.

**Table 5:** Overview of therapeutic strategies targeting Gal-3 in PDAC.

Therapeutic Strategy	Description	Ongoing Research and Clinical Efforts	Major Conclusions	References
Polysaccharide Inhibitors	Focuses on molecules like HH1-1 that hinder Gal-3 interactions and related signaling cascades.	Identification and optimization of molecules targeting Gal-3.	HH1-1 obstructs Galectin-3/EGFR/AKT/FOXO3 signaling, affecting various cellular activities.	[62]
Modified Citrus Pectin (MCP)	MCP challenges Gal-3’s dominance in the tumor microenvironment.	Preclinical testing for efficacy in mitigating tumor growth.	MCP holds potential in hampering tumor proliferation and metastasis.	[64]
Polysaccharides (e.g., RN1)	RN1, from Panax notoginseng, inhibits Gal-3 expression and disrupts its interactions.	Delve deeper into RN1’s interaction mechanisms and clinical efficacy.	RN1 disrupts various oncogenic pathways, highlighting its therapeutic potential.	[63]
Antibodies and RNA Interference	Precision-targeted strategies leveraging the specificity of antibodies and RNA interference mechanisms.	Refining molecular targets and delivery mechanisms.	Precision-based approach offers specificity against Gal-3.	[63]
Combination Therapies	Combining Gal-3 inhibitors with other treatments like immune checkpoint inhibitors or chemotherapy.	Investigating synergies and formulating clinical trial strategies.	Combination therapies have potential for amplified therapeutic outcomes.	[63]
Therapeutic Vaccines	Creating vaccines, like GCS-100, to stimulate immune response against Gal-3-presenting cells.	Development and optimization of Gal-3 targeted vaccines.	Vaccines can boost immune response leading to increased cytotoxic effects.	[80,81]

## Data Availability

This review article does not contain any new experimental data or datasets generated or analyzed during the current study. All data derived and discussed within this review are sourced from previously published articles, which are duly cited.
